# Transcriptional response of porcine skeletal muscle to feeding a linseed-enriched diet to growing pigs

**DOI:** 10.1186/s40104-016-0064-1

**Published:** 2016-02-08

**Authors:** Hongkui Wei, Yuanfei Zhou, Shuzhong Jiang, Feiruo Huang, Jian Peng, Siwen Jiang

**Affiliations:** Department of Animal Nutrition and Feed Science, College of Animal Science and Technology, Huazhong Agricultural University, Wuhan, 430070 P. R. China; Key Lab of Agricultural Animal Genetics, Breeding and Reproduction of Ministry of Education & Key Lab of Swine Genetics and Breeding of Ministry of Agriculture, Huazhong Agricultural University, Wuhan, 430070 P. R. China

**Keywords:** Linseed, mRNA expression, n-3 PUFAs, Skeletal muscle

## Abstract

**Background:**

To investigate the effect of feeding a linseed-enriched diet to growing-finishing pigs on gene expression in skeletal muscle, pigs were fed with a linseed-enriched diet for 0, 30, 60 and 90 d. Transcriptional profiles of *longissimus dorsi* muscle were measured using Affymetrix Genechip.

**Results:**

Results showed that 264 genes were identified as differentially expressed genes (DEGs). The strongest transcriptional response was clearly observed at 30 d. DEGs were assigned to several main functional terms, including transcription, apoptosis, intracellular receptor-mediated signaling, muscle organ development, fatty acid metabolic process, cell motion, regulation of glucose metabolic process, spermatogenesis and regulation of myeloid cell differentiation. We also found that transcriptional changs of several transcription cofactors might contribute to n-3 PUFAs regulated gene expression. In addition, the increased expression of *IGF-1*, insulin signaling pathway and the metabolism of amino acids might involve in the muscle growth induced by feeding a linseed-enriched diet. The results also provide the new evidence that the expression changes of *PTPN1*, *HK2* and *PGC-1α* might contribute to the regulation of insulin sensitivity by n-3 PUFAs.

**Conclusions:**

Our finding provided correlative evidence that feeding the linseed enriched diet affact expression of genes involved in insulin signaling pathway and the metabolism of amino acids.

## Background

Dietary n-3 polyunsaturated fatty acids (n-3 PUFAs) play an essential role in animal development and maintaining animal health. Ingestion of n-3 PUFAs will lead to their distribution to virtually every cell in the body with effects on neurological development, reproduction, insulin action, innate and acquired immunity [1]. Therefore, majority of the studies aimed at increasing the n-3 PUFAs levels in pork to improve its nutritional quality, by using the diet enriched in either fish oils [2], or plant sources of 18:3 such as linseed [3] and canola oil [4]. Interestingly, Sousa et al. [5] reported that feeding the pigs with a diet including 10 % linseed oil (α-linolenic acid, C18:3n-3) increased the lean tissue in pigs. Similarly, feeding pigs with a linseed-enriched diet was reported to result in a modest increase in skeletal muscle mass in pigs [6]. Moreover, it has been demonstrated that feeding a linseed-enriched diet increased the intramuscular fat content in porcine longissimus muscle [7].

The broad physiological impact of n-3 PUFAs are dependent on their biologically properties of altering membrane structure and function, enzymatic activities, eicosanoid signaling, as well as cellular interactions [8]. More recently, n-3 PUFAs have been demonstrated to influence the activation or expression of various transcription factors, including peroxisome proliferator activated receptors (PPARs), hepatic nuclear-4α (HNF-4α), nuclear factor κB (NF-κB), retinoid X receptor α (RXRα), sterol regulatory element binding protein-1c (SREBP-1c), and liver X receptors (LXR) [9]. Therefore feeding animals with an n-3 PUFAs enriched diet could make dramatic transcriptional changes in animal tissues.

Microarray technology has accelerated our ability to understand the transcriptomes in various species and tissues. Using microarray, several studies have addressed to elucidate the effect of dietary n-3 PUFAs on mRNA expression in central nervous system (CNS) [10], liver [11] and intestine [12]. In the current study, we characterized gene expression signatures in the *longissimus dorsi* muscle of pigs fed with a linseed-enriched diet for 0, 30, 60 or 90 d using Affymetrix Genechip.

## Methods

### Diets and animals

Two diets for growing pigs and two diets for finishing pigs were formulated to meet the NRC recommended requirements (1998): the control diets and the linseed-enriched diets containing 10 % linseed. Diets composition was presented in Table 1. The fatty acid composition of the diets was analyzed by gas chromatography and listed in Table 2. The linseed-enriched diet contained up to 30-fold more linoleic acid (C18:3 n3) than the control diet. In addition, 2-fold linoleic acid was observed in the linseed-enriched diet compared with the control diet. The control diet contained 6-fold more palmitic acid (C16:0) than the linseed-enriched diet.

**Table 1:** Composition and calculated analysis (as-fed basis) of diets

	Growing phase^a^		Finishing phase^b^	
Item	Control diet	Linseed-enriched diet	Control diet	Linseed-enriched diet
Ingredients, %				
Corn	48.70	60.50	52.90	65
Wheat middling	18.00			
Wheat bran			20.00	1.00
Soybean meal	27.00	26.50	21.00	21.00
Fat powder^c^	3.30		3.10	
linseed		10.00		10.00
Premix^d^	3.00	3.00	3.00	3.00
Calculated analysis				
CP, %	18.07	18.01	16.05	16.08
Ether extract, %	5.59	5.87	5.56	6.02
DE, Mcal/kg	3.42	3.41	3.40	3.40
Lysine, %	1.07	1.05	0.93	0.95
Calcium, %	0.69	0.66	0.65	0.70
Phosphorus, %	0.50	0.51	0.49	0.55

^a^Body weight range from 30 to 60 kg

^b^Body weight range from 60 to 115 kg

^c^Fat powder: The fat powder is commercially available (BERGAFAT HTL-306 from Berg & Schmidt Co.) with a main composition of a palm oil fraction and phospholipid and the ether extract content is more than 99.50 %. Fatty acid profile of the fat powder: palmitic acid (16:0), 70–80 %; stearic acid (18:0), 5–10 %; oleic acid (18:1), 8–15 %

^d^Provided per kg of premix: vitamin A, 11,250 IU; vitamin D_3_, 2,500 IU; vitamin E, 200 mg; menadione, 2.5 mg; thiamine, 2.5 mg; riboflavin, 6.0 mg; niacin, 25 mg; d-panthothenic acid, 8 mg; vitamin B_6_, 3.0 mg;vitamin B_12_, 0.08 mg; d-biotin, 0.1 mg; folic acid, 12.5 mg. copper, 20 mg; iron, 50 mg; manganese, 30 mg; zinc, 80 mg; iodine, 0.8 mg

**Table 2:** Fatty acid composition (g/100 g of total fatty acids) of diets

	Growing phase^a^		Finishing phase^b^	
Item	Control diet	Linseed diet	Control diet	Linseed diet
C14:0	1.09	0.11	1.10	0.11
C16:0	69.50	11.40	69.60	10.54
C16:1	0.10	0.38	0.11	0.39
C18:0	4.00	4.76	4.09	4.25
C18:1 n-9	11.73	24.81	10.74	24.18
C18:2 n-6	12.49	24.17	11.96	25.90
C18:3 n-3	0.97	30.34	1.08	32.59
C20:0	0.19	0.21	0.18	0.21
C20:1	0.06	0.06	0.04	0.23
C22:0	0.04	0.12	0.05	0.11
C22:6 n-3	0.03	0.39	0.09	0.20
SFA^c^	74.82	16.6	75.02	15.22
PUFAs^d^	13.49	54.9	13.13	58.69
n-3 PUFAs^e^	1.08	30.78	1.24	32.85
n-3/n-6 ratio^f^	0.09	1.27	0.1	1.27

^a^Body weight range from 30 to 60 kg

^b^Body weight range from 60 to 115 kg

^c^Saturated fatty acids percentage is sum of C14:0, C16:0, C18:0, C20:0 and C22:0

^d^Polyunsaturated fatty acids percentage is sum of C18:2 n-6, C22:6 n-3 and 18:3 n-3 (α-linolenic acid)

^e^n-3 polyunsaturated fatty acids (n-3 PUFAs) percentage is the sum of C18:3 n-3 (α-linolenic acid) and C22:6 n-3

^f^n-3/n-6 ratio was calculated as the ratio between the total n-3 PUFAs and the total n-6 PUFAs

Twenty-four 80 d age barrows (Landrace × Yorkshire, Huazhong Agricultural University) weighing 35 ± 3.7 kg were randomly assigned to 1 of 4 groups. Pigs in those 4 groups were fed with linseed diet for 0, 30, 60, 90 d before slaughter, respectively. The 0 d and 90 d groups were fed with the control or linseed growing diets for 30 d and then fed with the corresponding finishing diets for the following 60 d. Following feeding the control growing diet for 30 d and control finishing diet for 30 d, pigs in the 30 d group were fed with the linseed finishing diet for 30 d before slaughter. Following feeding the control growing diet for 30 d, pigs in the 60 d group were fed with the linseed finishing diet for 60 d before slaughter. Throughout the experimental period, pigs were housed individually and fed ad libitum during the trial period. At the age of 170 d, all the pigs were humanely slaughtered after 24 h fasting. The *longissimus dorsi* muscle samples were rapidly removed. Following removing the intermuscular adipose tissue, the muscle samples were frozen in liquid nitrogen to be stored at −80 °C. The study was carried out according to Huazhong Agriculture University Animal Care and Use Committee guidelines.

### RNA extraction

RNA samples from three biological replicates for every treatment were extracted using TRIzol (Invitrogen Corp., Carlsbad, CA, USA) as described by the manufacturer, followed by DNase digestion using DNAfree kit (Applied Biosystems, Foster City, CA, USA) according to the manufacturer’s instructions and quantitated by spectrophotometric (Eppendorf, Hamburg, Germany) absorbance at 260 nm. RNA quality was evaluated using A260/A280 ratio (>1.8) and the RNA 6000 Nano Chips in the Agilent 2100 Bioanalyzer (Agilent Technologies, Palo Alto, CA, USA).

### Microarray hybridization and analysis

Hybridisation with Affymetrix GeneChip® Porcine Genome Arrays was performed at CapitalBio Corporation (Beijing, China). The model-based expression index algorithm implemented in dCHIP software was used for data pre-processing [13]. Probe sets were filtered using two criteria: (a) P call rate in arrays ≥20 % and (b) high-intensity (≥55) rate in arrays ≥75 %. The extraction and analysis of differential gene expression algorithm [14] was used for differential gene expression analysis. Q < 0.05 was selected to designate differentially expressed genes [15].

Affymetrix Porcine Annotation V5 [16] was used to annotate the differentially expressed genes. Genes represented by multiple probe sets were determined based on previous studies [17]. Briefly, if a gene is represented by a _at probe set and multiple _s_at probe sets or _x_at probe sets simultaneously, the _at probe set is selected. We filtered the genes that exhibited multiple expression patterns at the probe set level. The average expression value was then calculated to represent the expression of probe sets. After removing the represented probe sets, cluster analysis was performed using the self-organizing tree algorithm (SOTA) implemented in Multiple Array Viewer software V.4.81 [18]. We selected the candidate genes involved in muscle mass regulation from the differentially expressed genes as follows. We constructed a synthesized gene set containing genes related to muscle mass regulation based on gene ontology (GO) annotation and literature mining. Pubgene [19] and GOGene [20] are literature mining tools that can retrieve genes involved in skeletal muscle growth in our data. Database for Annotation, Visualization and Integrated Discovery software [21] was used to annotate and analyze GO and pathway.

### Quantitative -PCR

Synthesis of cDNA from skeletal muscle was performed as previously described [22]. Differential expression was checked by Q-PCR for some genes, e.g. *peroxisome proliferators-activated receptor-γcoactivator 1α* (*PGC-1α*), *protein tyrosine phosphatase non-receptor type 1* (*PTPN1*), *baculoviral IAP repeat-containing protein 2* (*BIRC2*)*, insulin-like growth factor 1* (*IGF-1*)*, Myocyte-specific enhancer factor 2C* (*MEF2C*) and *signal transducer and activator of transcription 5* (*STAT5A*). The primers were designed using Primer5 software. Relative mRNA levels of genes were quantified using Q-PCR with an iQ™127 5 Real Time PCR Detection System (BioRad, Hercules, CA, USA). The *β-actin* mRNA levels were similarly measured and served as the reference gene. Of each cDNA, 0.1 μg were added to Q-PCR reagent mixture, SYBR® qPCR Mix (Toyobo, Fukui, Japan), with the sense and antisense primers (500 nmol/L each). The PCR parameters were as follows: denaturation at 95 °C for 3 min followed by 40 cycles of denaturation at 94 °C for 20 s, annealing for 20 s, and extension at 72 °C for 20 s and reading plate for 10s. All sample mRNA levels were normalized to the values of *β-actin* and the results expressed as fold changes of threshold cycle (Ct) value relative to controls using the 2^-ΔΔCt^ method [23]. All samples were measured in triplicate.

## Results

### Response of global transcriptome in longissimus dorsi muscle to feeding a linseed-enriched diet to growing pigs

The analysis revealed that 368 probe sets were differentially expressed through the course of trial period (Q < 0.05). After removing the redundant probe sets, 264 probe sets were remained. Among these 264 probe sets, 244 (92.4 %) were assigned to known genes. For each DEGs, the maximal FC between the different time point was larger than 1.5. Additionally, the expression of 87 DEGs (29.5 %) were changed ≥ 2.0-fold throughout the course of trial period. The top 10 up-regulated and down-regulated genes were listed in Tables 3 and 4.

**Table 3:** Top 10 up-regulated genes

Gene	Product	Log intensity^a^				Maximal FC^b^
		0 d^c^	30 d	60 d	90 d	
*RSAD2*	Viperin	6.151	6.52	8.01	6.39	6.28
*HLA-A3*	HLA class I histocompatibility antigen, A-3 alpha chain precursor	7.47	9.46	8.16	6.73	4.58
*PGC-1α*	Peroxisome proliferators-activated receptor-γcoactivator 1α	6.95	7.69	7.18	8.99	4.24
*NNAT*	Neuronatin	6.51	7.41	8.03	8.16	3.25
*PTGES3*	Telomerase-binding protein p23	8.94	8.36	10.01	8.68	3.14
*KCNQ5*	Potassium voltage-gated channel subfamily KQT member 5	4.80	6.20	4.81	4.58	3.06
*SNX21*	Sorting nexin 21	6.54	6.03	7.30	7.57	3.01
*HK2*	Hexokinase, type II	7.34	8.74	8.29	8.10	2.70
*PAF1*	Polymerase associated factor	7.52	8.77	7.49	7.65	2.44
*TRIM35*	Tripartite motif-containing 35 isoform 2	5.42	6.61	5.64	5.41	2.41

^a^Log intensity is calculated from Log_2_ (normalized intensity)

^b^Maximal FC (Fold change) is calculated from the ratio of maximum intensity/minimum intensity among the time points

^c^Pigs in 0 d, 30 d, 60 d and 90 d groups (80 d age) were first fed with the control diet for 90, 60, 30 and 0 d and then fed the linseed diet for 0, 30, 60 and 90 d, respectively. At 110 d of age, pigs switched from grower to finisher diet

**Table 4:** Top 10 down-regulated genes

Gene	Product	Log intensity^a^				Maximal FC^b^
		0 d^c^	30 d	60 d	90 d	
*FAM134B*	Family with sequence similarity 134, member	9.97	8.75	9.54	10.78	−3.91
*SLC19A2*	Thiamine transporter 1	8.34	7.71	8.29	9.50	−3.71
*CREM*	CAMP responsive element modulator	9.18	8.57	9.09	10.38	−3.69
*FAM13A1*	Protein FAM13A1	9.08	7.79	8.92	9.39	−2.98
*SEMA6A*	Semaphorin 6A precursor	7.11	5.96	7.55	7.09	−2.97
*TNFRSF12A*	Tumor necrosis factor receptor superfamily member Fn14 precursor	11.01	9.87	10.78	11.40	−2.93
*SDC2*	Syndecan-2 precursor	8.33	7.42	8.60	8.84	−2.66
*TRAC*	T-cell receptor alpha chain C region	6.27	4.88	6.20	5.98	−2.62
*ABRA*	Striated muscle activator of Rho-dependent signaling	11.78	10.96	11.71	12.33	−2.61
*NQO1*	NAD(P)H dehydrogenase	10.25	8.86	9.77	9.62	−2.55

^a^Log intensity is calculated from Log_2_ (normalized intensity)

^b^Maximal FC (Fold change) is calculated from the ratio of maximum intensity/minimum intensity among the time points

^c^Pigs in 0 d, 30 d, 60 d and 90 d groups (80 d age) were first fed with the control diet for 90, 60, 30 and 0 d and then fed the linseed diet for 0, 30, 60 and 90 d, respectively. At 110 d of age, pigs switched from grower to finisher diet

**Fig. 1:**
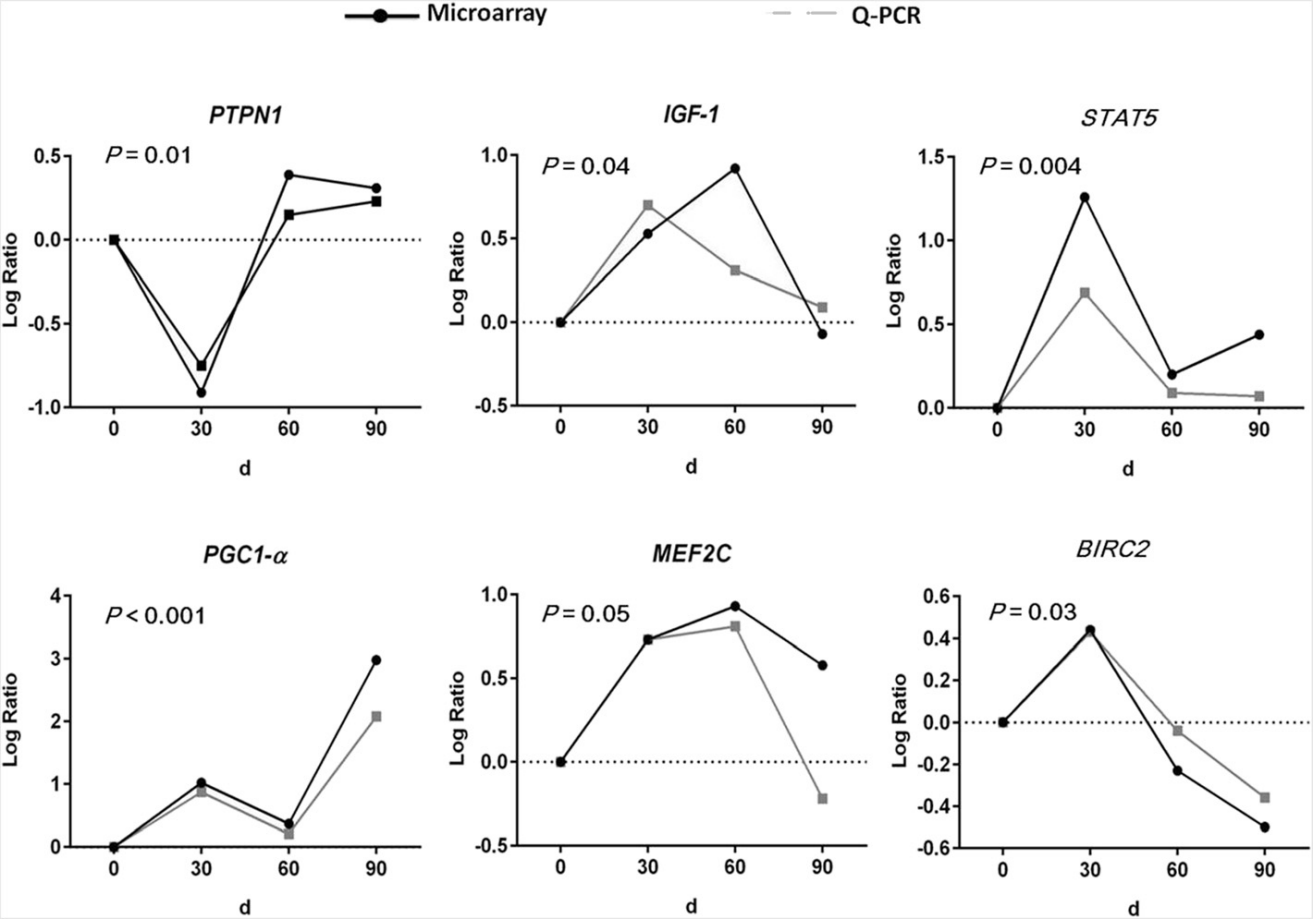
Validation of differentially expressed genes by q-PCR. Pigs in 0 d, 30 d, 60 d and 90 d groups (80 d age) were first fed with the control diet for 90, 60, 30 and 0 d and then fed the linseed diet for 0, 30, 60 and 90 d, respectively. At 110 d of age, pigs switched from grower to finisher diet. Transcriptional profiles of *longissimus dorsi* muscle were measured using Affymetrix Genechip. Data from q-PCR and microarray were transformed to LogRatio value. The LogRatio value was the ratio of logarithm (base 2) of the mean value of the time point to the mean value of 0 d level. *P*-values were calculated from q-PCR results

In order to validate the results of microarray, six genes (*PTPN1*, *PGC-1α*, *BIRC2*, *MEF2C*, *IGF-1* and *STAT5*) were chosen for Q-PCR confirmation. Comparison of the Q-PCR results with the microarray data demonstrated that these genes had statistically significant expression patterns which were similar to those seen in the microarray data (Fig. 1), indicating that our microarray data are highly reliable and accurate.

### Clustering of DEGs

**Fig. 2:**
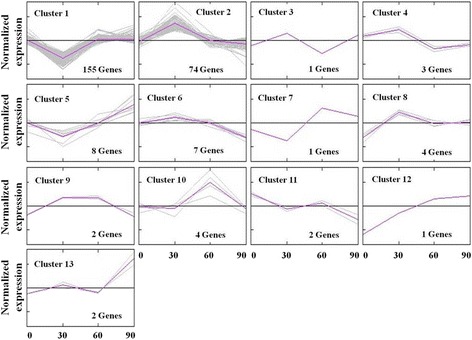
Clustering of differentially expressed genes. Pigs in 0 d, 30 d, 60 d and 90 d groups (80 d age) were first fed with the control diet for 90, 60, 30 and 0 d and then fed the linseed diet for 0, 30, 60 and 90 d, respectively. At 110 d of age, pigs switched from grower to finisher diet. Transcriptional profiles of *longissimus dorsi* muscle were measured using Affymetrix Genechip. SOTA algorithm was performed for DEGs clustering analysis. Each gene was normalized around zero and then clustered using self-organizing tree algorithm. The purple line was the average expression pattern

To extract clearer and meaningful expression patterns, we performed SOTA algorithm for DEGs clustering analysis. The results showed that the DEGs were assigned to 13 clusters (Fig. 2). Interesting, most DEGs (229) were assigned to two main clusters. In cluster 1, there was 155 (58 %) DEGs, of which the expression level was significantly down-regulated at 30 d but raised their expression at 60 and 90 d comparable to those at 30 d. The cluster 2, which contained 74 (28 %) DEGs, had a inverse expression pattern to that of the cluster 1, that genes were significantly induced at 30 d but recovered at 60 and 90 d. The numbers of gene in other clusters except cluster 1 and cluster 2 were all below of 10. DEGs in the cluster 8, cluster 10 and cluster 12 were up-regulated throughout the trail period. These results showed that a strong transcriptional response was clearly observed at 30 d (Fig. 2).

### Go and pathway analysis of DEGs

To understand the biological functions of the DEGs, GO and pathway analysis were performed in the current study. The results showed that a significant number (*p* < 0.05) of DEGs were annotated as being involved in transcription (32 genes), apoptosis (21 genes), intracellular receptor-mediated signaling (7 genes), muscle organ development (10 genes), fatty acid metabolic process (9 genes), cell motion (15 genes), regulation of glucose metabolic process (4 genes), spermatogenesis (9 genes) and regulation of myeloid cell differentiation (5 genes) (Table 5).

**Table 5:** GO (gene ontology) enrichment of differentially expressed genes

Category	Count	*P* Value^a^
Transcription, DNA-dependent	32	0.001
Apoptosis	21	0.002
Intracellular receptor-mediated signaling	7	0.003
Muscle organ development	10	0.011
Fatty acid metabolic process	8	0.017
Cell motion	15	0.021
Regulation of glucose metabolic process	4	0.025
Spermatogenesis	9	0.035
Regulation of myeloid cell differentiation	5	0.035

^a^ *P* value was calculated by hypergeometric distribution test. Enrichment was considered significant at *P* < 0.05

Pathway analysis revealed that a total of 9 pathways were above the significance threshold (Q < 0.01), including regulation of actin cytoskeleton, amino acids metabolism, biosynthesis of steroids, IκB kinase NF − κΒ cascade related genes, cell adhesion molecules (CAMs), IGF-1/insulin signaling pathway, ubiquitin mediated proteolysis, Wnt signaling pathway and neuroactive ligand-receptor interaction (Table 6). We highlighted the interested GO and pathways with the associated genes as following.

**Table 6:** Pathway enrichment of differentially expressed genes

Pathway	Count	Q-Value^a^
Regulation of actin cytoskeleton	7	0.000
Amino acids metabolism	3	0.001
Biosynthesis of steroids	3	0.002
IκB kinase NF-κB cascade related genes	6	0.003
Cell adhesion molecules (CAMs)	4	0.008
Insulin signaling pathway	5	0.008
Ubiquitin mediated proteolysis	4	0.008
Wnt signaling pathway	5	0.010
Neuroactive ligand-receptor interaction	5	0.010

^a^Q value was calculated by hypergeometric distribution test. Enrichment was considered significant at Q < 0.01

### Muscle organ development

The expression of other genes involved in regulation of muscle organ development were also altered, including *MEF2C*, *COUP transcription factor 2* (*NR2F2*) and *Zinc finger protein GLI1* (*Gli1 leptin receptor*) et al. Our results showed a significant increase in mRNA abundance of *MEF2c* and *NR2F2*, but a significant decrease in *Gli1* mRNA abundance after feeding a linseed-enriched diet for 30 d.

### Fatty acid metabolic process

Eight genes involved in fatty acid metabolic process were changed their mRNA expression level in response to feeding a linseed-enriched diet. The expression of *fatty acid synthase* (*FASN*), elovel fatty acid elongase 6 (*ELOVL6*), *Estradiol 17-beta-dehydrogenase 1* (*HSD17B1*) and mevalonate kinase (*MVK*) was decreased after feeding pigs with a linseed-enriched diet for 30 d. However, the expression of *glycerol-3-phosphate acyltransferase 1, mitochondrial* (*GPAM*) and *prostaglandin-endoperoxide synthase 2* (*PTGS2*) was increased at 30 d. In addition, feeding pigs with a linseed-enriched diet induced the expression of *PGC-1α* at 30 and 90 d. Moreover, the expression intensity of *prostaglandin E synthase 3* (*PTGES3*) was decreased from 8.9 to 8.4 at 30 d, but then raised to 10.0 at 60 d.

### Regulation of glucose metabolic process

Three genes (*HK2*, *PGC-1α* and *ALDOC*) involved in regulating glucose metabolic process were differently expressed in the current study. The expression of *HK2* was induced 2.4-fold at 30 d and remained up-regulated at the later time points. For *ALDOC*, feeding a linseed-enriched diet inhibited its expression at 30 d.

### Amino acids metabolism

Notrworthly, expression of amino acids metabolism related genes were also influenced by feeding pigs with a linseed-enriched diet. For example, expression of *solute carrier family 38 member 1* (*SLC38A1*), which is an amino acid transporter, and glutamate-cysteine ligase catalytic subunit (GCLC), were up-regulated at 30 d, while *arginase type 2*, a gene involved in arginine catabolism, was down-regulated at 30 d.

### IκB kinase NF − κΒ cascade

Concerning the top one enriched pathway, the IκB kinase NF-κB cascade, 6 genes were identified as candidate genes. Among them, 4 stimulators of the IκB kinase NF-κB cascade *solute carrier family 20, member 1* (*SLC20A1*), *TRAF3 interacting protein 2* (*TRAF3IP2*), *tumor necrosis factor receptor superfamily member Fn14 precursor* (*TNFRSF12A*), and *toll-like receptor 9 precursor* (*TLR9*), were down-regulated at 30 d (fold change ≥1.8). *BIRC2*, an inhibitor of IκB kinase, was up-regulated at 30 d with a 1.7-fold expression change. Interestingly, *Tumor protein p53 inducible nuclear protein 1* (*TP53INP1*), another stimulator of the IκB kinase NF-κB cascade, was down-regulated at 90 d with a 2-fold expression change.

### IGF-1/insulin signaling

Four genes involved in IGF-1/insulin signaling were differentially expressed. Among them, STAT5, a transcription factor involved in growth hormone (GH) signaling, was up-regulated in response to feeding pigs with a linseed-enriched diet at 30 d. In addition, significantly lower level of *PTPN1*, a gene involved in regulation of GH signaling and tyrosine kinase receptor signaling was detected after feeding pigs with a linseed-enriched diet at 30 d. However, both *PGC-1α* and sorbin and SH3 domain-containing protein 1 (*SORBS1*)*,* the positive regulators of insulin signaling, were up-regulated at 30 d.

### Wnt signaling pathway

The results from the current study suggested that feeding pigs with a linseed enriched-diet influenced the mRNA expression of several genes coding the elements in Wnt pathway, including *FBXW11*, *APC*, *LEF1*, *LDB1*, *MED1* and *KAT5*. The expression of *FBXW11* and *KAT5* in porcine skeletal muscle was increased after feeding a linseed-enriched diet for 30 d. However, the expression of *APC*, *LEF1* and *LDB1* was decreased at 30 d.

## Discussion

It is well known that n-3 PUFAs play a key role in CNS development and liver metabolism by regulating of genes expression [10, 11]. To investigate the effect of dietary n-3 PUFAs on gene expression in skeletal muscle, we focused on the whole transcriptional response to feeding pigs with a linseed-enriched diet in porcine skeletal muscle in the present study.

Several studies have shown that n-3 PUFAs can act as ligands to influence gene expression by regulation of activation or/and expression of transcription factors [9]. In the current study, we showed that 32 (13.1 %) DEGs were related to gene expression regulation (Table 4). These 32 DEGs contained not only transcriptional factors, such as *LEF1*, but also transcriptional coactivator and corepressor, i.e., *PGC-1α*, *LDB1*, *SIRT1*, *SP100*, *NR2F2*, *ABRA*, *HTATIP* and *ARNT*, suggesting that the transcription cofactors might also contribute to n-3 PUFAs regulated gene expression.

In the past decade, dietary n-3 PUFAs has been demonstrated to influence energy metabolism in skeletal muscle, in part by acting on PPARs [24]. In the current study, the GO analysis of the DEGs showed that feeding pigs with a linseed-enriched diet significantly influence the glucose metabolic process. Both *HK2* and *PGC-1α* were in the top 10 up-regulated genes list, suggesting a remarkable change of glucose metabolism might be induced in skeletal muscle by feeding pigs with a linseed-enriched diet. The expression of *HK2* was induced 2.4-fold at 30 d and remained up-regulated at the later time points. A hexokinase is an enzyme that phosphorylates hexoses to form hexose phosphate. In most organisms, glucose is the most important substrate of hexokinases, while glucose-6-phosphate is the most important product [25]. Hexokinase has three different isoforms. In muscle cells, HK2 is the predominant isoform [26]. For the significantly decreased activation and expression of HK2 in type II diabetes, it was investigated as a promising candidate gene for type II diabetes.

PGC-1α is another up-regulated gene involved in regulating of glucose metabolic process in the current study. It was a later-induced gene, whose expression was increased 4.3-fold at d 90. PGC-1α is a transcriptional coactivator with an important role in regulation of energy homeostasis [27]. In skeletal muscle, PGC-1α promotes glucose uptake and expression of genes involved in the electron transport chain and oxidative phosphorylation [28]. Therefore our finding provide new evidence that diet n-3 PUFA may improve insulin sensitivity in skeletal muscle.

Our result showed that feeding pigs with a linseed enriched diet influenced the expression of 8 genes involved in fatty acids metabolic process, including *PTGES3*, *GPAM*, *PTGS2*, *FASN*, *HSD17B1*, *MVK*, *ELOVL6* and *PGC-1α*. The main function of fatty acid synthase is to catalyze the synthesis of palmitate from acetyl-CoA and malonyl-CoA. Elongation of long chain fatty acids 6 (ELOVL6) is an elongase that catalyzes the conversion of palmitate to stearate [29]. In the current study, the decreased expression of *FASN*, the gene coding fatty acid synthase, and *ELOVL6* implicated that feeding pigs a linseed-enriched diet inhibit the synthesis of palmitate and stearate in skeletal muscle. In addition, the increased expression of *GPAM* at 30 d suggested an increased biosynthesis of phospholipid. Moreover, the expression changes of *PTGES3*, *HSD17B1* and *MVK* indicated that feeding pigs with a linseed-enriched diet might influence the biosynthesis of steroids. Although the intermuscular adipose tissue was removed from muscle samples in the current study, the skeletal muscle also contains the intramuscular fat. That might be why *FASN*, a highly expressed gene in adipose tissue, was detected in the current study.

It is also of interest to note that mRNA abundance of several genes involved in amino acids metabolism was altered. SLC38A1 is a transporter of neutral amino acids, including glycine, alanine, serine, cysteine, glutamine, asparagine, histidine, methionine, threonine, proline, tyrosine and valine [30]. Therefore the increased expression of *SLC38A1* at 30 d in the current study might result in the increased uptake of amino acid in muscle cells. Additionally, feeding pigs with a linseed-enriched diet decreased the expression of *ARG2*, a gene involved in arginine catabolism. This result provide new evidence suggesting that feeding pigs with a n-3 PUFA enriched diet decrease the whole-body amino acids oxidation [31]. Moreover, the increased expression of *GCLC*, a rate limiting enzyme in glutathione synthesis, suggested that feeding pigs with a linseed-enriched diet might increase the glutathione level in porcine skeletal muscle. These results suggested that feeding pigs with a linseed-enriched diet might increase the available amino acids in skeletal muscle, especially for arginine and glutathione.

Arginine has been demonstrated to promote skeletal muscle growth by generation of nitric oxide and/or activation of mTOR Signaling [32]. Zheng et al. reported that there is a positive correlation between the expression of *GCLC* and the postnatal growth rate of skeletal muscle [33]. Therefore the expression changes of *GCLC* and *ARG2* might contribute to the growth of skeletal muscle induced by feeding pigs with a linseed-enriched diet. Actually, we have previously reported that muscle mass in pigs fed a linseed-enriched diet is increased [6].

The expression of several genes involved in muscle organ development was changed in the current study. MEF2c and NR2F2 are well characterized to be essential for myoblast differentiation [34, 35], whereas, Gli1 blocks myoblast differentiation by inhibiting MyoD activation in skeletal muscle [36]. In the current study, the increased expression of *MEF2c* and *NR2F2* and a decreased expression of *Gli1* at d 30 suggested that feeding pigs with a linseed-enriched diet might increase the differentiation of myogenic cells in porcine skeletal muscle. However, further studies are needed to confirm these results.

IGF-1 is essential for normal growth in numerous mammalian. Although more than 80 % IGF-1 appearing in the circulation is produced by liver, locally produced IGF-1 in muscle is more important than circulating IGF-1 in controlling muscle growth [37]. In the current study, the pigs fed the linseed-enriched diet for 30 and 60 d had a higher expression of *IGF-1* in porcine skeletal muscle than those only fed the control diet, suggesting that feeding a linseed-enriched diet may improve the growth of skeletal muscle.

We also showed that feeding pigs with linseed-enriched diet increased mRNA expression of *STAT5A*, an essential mediator of GH-induced *IGF-1* expression in skeletal muscle [38]. In addition, PTPN1 attenuates growth hormone-mediated JAK2-STAT5 signaling [39], and thus, the suppressed *PTPN1* expression might also contribute to the increased *IGF-1* expression in this study. In the present study, the decreased expression of *PTPN1* corresponded to another microarray study, where a significant inhibition of *PTPN1* expression is detected in liver of mice fed an n-3 PUFAs-enriched diet compared with those fed an n-6 PUFAs-enriched diet [11].

PTPN1 can also act as a key negative regulator of insulin signaling by dephosphorylation of IR and IRS-1 [40]. In addition, the expression of two positive regulators of insulin signaling, *PGC-1α* and *SORBS1*, was increased at 30 d and/or 90 d. It has been demonstrated that insulin could stimulate skeletal muscle protein synthesis by activating of mTOR signaling pathway in young animals [41]. Therefore those results suggested that feeding pigs with a linseed-enriched diet might increase the insulin-induced protein synthesis in skeletal muscle. This hypothesis is in coincidence with a previous study, in which increased insulin response is associated with higher muscle activation of mTOR pathway and higher whole-body disposal of amino acids in growing bovine fed with a n-3 PUFA-enriched diet [42].

Wnt signaling pathway is implicated in the control of cell proliferation and differentiation. When Wnt signaling is turned on, it will promote the proliferation and inhibit the differentiation, otherwise the proliferation will be inhibited [43]. In the canonical Wnt pathway, Wnt binding leads to the stabilization of the transcription factor β-catenin, which enters the nucleus to active LEF1 and regulates the expression of Wnt pathway target genes. LDB1, MED1 and KAT5 are transcriptional coactivators of Pitx2, which is a target gene of LEF1 and plays an important role in regulation of cell proliferation. APC is a blocker of Wnt signaling which can stabilize the β-catenin complex, while FBXW11 can targets phosphorylation-dependent degradation of β-catenin [44]. In the current study, the increased expression of *FBXW11* and decreased expression of *LDB1* and *MED1* indicated that feeding pigs with a linseed-enriched diet might increase the degradation of β-catenin and inhibit the Pitx2-induced cell proliferation. However, the results of the decreased expression of *APC* and the increased expression of KAT5 did not support the inference that activation of Wnt signaling pathway was decreased in the current study. The controlled studies are needed to explore whether n-3 PUFA inhibit the activation of Wnt signaling pathway.

In the current study, we showed that linseed-enriched diet up-regulated the expression of genes (*SLC20A1*, *TRAF3IP2*, *TP53INP1*, *TNFRSF12A*, *TLR9* and *BIRC2*) which involved in IκB inhibition in porcine skeletal muscle, and might lead to decrease NF-κB activation (Table 5). Recent studies indicated that activation of NF-κB is inhibited by n-3 PUFAs in lymphomonocyte [45], and the decreased IKK activation is likely to contribute to the suppressed NF-κB activation [46]. Our results provided an alternative explanation why n-3 PUFAs could suppress NF-κB activation.

NF-κB is a transcription factor that play important role in regulation of inflammatory processes. The expression of many pro-inflammatory cytokines such as *IL-1*, *IL-6* and *TNF-α*, are induced by NF-κB activation in various tissues [47]. Moreover, NF-κB mediates the anti-inflammation properties of n-3 PUFA partially [48]. Although our microarray results implicated that feeding pigs with a linseed-enriched diet decreased the activation of NF-κB, the expression of *IL-1*, *IL-6* and *TNF-α* were unfortunately not detected in our microarray experiment and thus filtered for further analysis. This might be cause by low expression level of these genes in skeletal muscles and low sensitivity of microarray. But it is previously reported that expression of *IL-1*, *IL-6* and *TNF-α* in skeletal muscle were inhibited by feeding a linseed-enriched diet using transcriptase polymerase chain reaction technique to assess the samples in this study [22].

The previous study showed that, by inhibiting the activation of NF-κB, EPA (20C: 5 n3) decreases the expression of *muscle RING-finger protein-1* (*MuRF1*), which plays an important role in controlling protein degradation in skeletal muscle [49]. Unfortunately, we did not find any oligonucleotide set representing *MuRF1* gene on the chip used in the current study. Interestingly, *PTPN1* has been shown to act as a target gene of NF-κB. Its expression level can be induced by NF-κB activation [50]. Therefore the decreased expression of *PTPN1* might cause by the inhibited activation of NF-κB.

## Conclusion

Genome-wide investigation of transcriptional response to feeding pigs with a linseed-enriched diet in porcine skeletal muscle provides systematic information for understanding the effect of dietary n-3 PUFA on the gene expression in skeletal muscle. Our finding suggested that the increased expression of *IGF-1*, insulin signaling pathway and the metabolism of amino acids might involve in the muscle growth induced by feeding a linseed-enriched diet. Our results also provide the new evidence that the expression changes of *PTPN1*, *HK2* and *PGC-1α* might contribute to the regulation of insulin sensitivity by n-3 PUFAs. However, the effect of dietary n-3 PUFA on the activation of signaling pathways needs to be confirmed by protein and enzyme activity studies.

## Abbreviations

BIRC2: baculoviral IAP repeat-containing protein 2
CAMs: cell adhesion molecules
CNS: central nervous system
DEGs: differentially expressed genes
ELOVL6: elovel fatty acid elongase 6
FASN: fatty acid synthase
GH: growth hormone
GO: gene ontology
GPAM: lycerol-3-phosphate acyltransferase 1, mitochondrial
HNF-4α: hepatic nuclear-4α
HSD17B1: Estradiol 17-beta-dehydrogenase 1
IGF-1: insulin-like growth factor 1
LXR: liver X receptors
MEF2C: Myocyte-specific enhancer factor 2C
MVK: mevalonate kinase
n-3 PUFAs: n-3 polyunsaturated fatty acids
NF-κB: nuclear factor κB
NR2F2: COUP transcription factor 2
PGC-1α: peroxisome proliferators-activated receptor-γcoactivator 1α
PPARs: peroxisome proliferator activated receptors
PTGES3: prostaglandin E synthase 3
PTGS2: and prostaglandin-endoperoxide synthase 2
PTPN1: protein tyrosine phosphatase non-receptor type 1
RXRα: retinoid X receptor α
SLC20A1: solute carrier family 20, member 1
SREBP-1c: sterol regulatory element binding protein-1c
STAT5A: signal transducer and activator of transcription 5
SORBS1: orbin and SH3 domain-containing protein 1
SOTA: self-organizing tree algorithm
TNFRSF12A: tumor necrosis factor receptor superfamily member Fn14 precursor
TLR9: toll-like receptor 9 precursor
TP53INP1: Tumor protein p53 inducible nuclear protein 1
TRAF3IP2: TRAF3 interacting protein 2
